# SQL on FHIR - Tabular views of FHIR data using FHIRPath

**DOI:** 10.1038/s41746-025-01708-w

**Published:** 2025-06-09

**Authors:** John Grimes, Ryan Brush, Nikolai Rhyzhikov, Piotr Szul, Joshua Mandel, Dan Gottlieb, Grahame Grieve, Bashir Sadjad, Arjun Sanyal

**Affiliations:** 1 Australian e-Health Research Centre, CSIRO Health and Biosecurity, Brisbane, Australia; 2 Google Health, Seattle, WA USA; 3 Health Samurai, Lisbon, Portugal; 4 Microsoft Research, Redmond, WA USA; 5 Department of Biomedical Informatics, Harvard Medical School, Boston, MA USA; 6 Central Square Solutions, Boston, MA USA; 7 Health Intersections, Melbourne, VIC Australia; 8 Google, Waterloo, ON Canada; 9 Antidote Solutions, Lancaster, PA USA

**Keywords:** Data integration, Data processing, Standards

## Abstract

Challenges exist with the adoption of Fast Healthcare Interoperability Resources (FHIR) within analytics, including the difficulty in transforming complex data structures, and performance issues when querying large datasets in their native JSON or XML formats. In 2023, an international working group began work on a solution to this problem that would be easier to implement than existing approaches. Over the course of 18 months, the group authored a new specification and validated it through the development and testing of multiple independent implementations. The outcome of this work is a standard, implementation-agnostic method for defining views that produce tabular data from FHIR resources. SQL on FHIR view definitions can be written to cover common use cases and can be executed across a variety of technology platforms. We evaluate the feasibility of this approach by replicating findings from an existing study across multiple view runner and database implementations, demonstrating portability and consistency.

## Introduction

FHIR (Fast Healthcare Interoperability Resources) is a modern healthcare interoperability standard that provides a structured way to exchange clinical and administrative health data. Evolving from predecessors such as HL7 v2 or v3, FHIR adopts a modular, resource-based approach where health information is represented as discrete, reusable components called resources. These resources (e.g., Patient, Observation, Medication) model different healthcare concepts and can be linked together to represent complex clinical scenarios.

While other widely used standards like OMOP CDM (Observational Medical Outcomes Partnership Common Data Model) focus on standardising data for research and analytics^1^, FHIR’s primary focus is on real-time data exchange and interoperability. FHIR resources are more granular and flexible than OMOP’s tables, supporting both REST (Representational State Transfer)-based APIs (Application Programming Interfaces) and bulk data operations. Each FHIR resource follows a consistent structure with standardised data types and can be extended through profiles to meet specific implementation needs.

FHIR has become increasingly common worldwide, being rapidly adopted by healthcare providers, research organisations, and software vendors^2^. Its implementation-friendly design, comprehensive documentation, and active community support have contributed to this widespread adoption.

Major regulatory bodies and health organisations are mandating or recommending FHIR as their preferred standard for healthcare data exchange. In the United States, the Office of the National Coordinator for Health Information Technology (ONC) has mandated FHIR API implementation through the 21st Century Cures Act^3^. The European Union has determined that FHIR will be the preferred interoperability standard within the European Health Data Space (EHDS)^4^, an initiative designed to enhance personal access to health data while also enabling secondary use for research and policy purposes^5,6^.

The increased availability of FHIR is driving its use for analytic use cases, including clinical decision support, predictive analytics and real-time alerting^7–11^. As this community of users grows, demand increases for standard tools to assist with the tasks of (1) converting heterogeneous healthcare data into FHIR format^12–14^; and (2) transforming FHIR data into structures more amenable to analytics.

FHIR is usually exchanged using JSON (JavaScript Object Notation) or XML (Extensible Markup Language) documents, containing nested data elements and governed by defined semantics concerning references between resources, data types, terminology, extensions, and other aspects of the specification.

To prepare FHIR data for analytic use cases, it is often necessary to transform it away from its JSON or XML form. Creation of use case-centric, tabular views of FHIR data is often desirable for the following reasons:

- Tabular data may be easier to visualise and understand for many users.
- Tabular data is compatible with a wider range of analytic tools, many of which are designed to work with flat, two-dimensional data structures.
- Tabular data can be more efficient to process and query than nested JSON or XML documents.

Writing code to extract tabular data from JSON or XML documents can be complex and error-prone, especially for users who are not familiar with the FHIR specification. In the absence of standard tools to support this work, a proliferation of independently developed transformation code can occur. This can, in turn, lead to duplication of effort and inconsistency in the data produced.

The original specification that this paper lends its name from is SQL on FHIR^15^, which is a standard method for querying FHIR data using SQL (Structured Query Language). The approach of the original specification was to retain the nested structure of FHIR data and use SQL to query it directly.

Several other initiatives have also been conceived, taking different approaches to the same problem. The FHIR Mapping Language^16^ was created within the FHIR specification to solve the problem of mapping between FHIR and other arbitrary data structures. Grimes et al.^17^ proposed a method for defining tabular views over FHIR data using the FHIRPath expression language. This method was implemented as part of the Pathling implementation, as an operation named ‘extract’. Commercial FHIR vendor, Firely, proposed a query language^18^ designed to project and filter FHIR data into a tabular result, agnostic of the storage implementation.

Despite this work, a significant research gap remains in the standardisation of FHIR data transformation for analytics. The existing approaches have been difficult to apply at scale due to either excessive complexity or limited portability across platforms. This paper addresses this gap by examining whether a constrained, deliberately simplified approach to FHIR data transformation can achieve sufficient expressivity while maintaining practical implementation characteristics across diverse technology platforms.

Specifically, we investigate how a specification focused exclusively on the transformation of FHIR resources to tabular structures can balance competing requirements of simplicity, expressiveness, and portability. Our research question asks: Can a view definition specification with deliberate constraints on scope and expressivity achieve practical implementation across diverse technology stacks while supporting real-world analytical use cases?

The paper documents the development and validation of the view definition specification, including demonstration of real-world portability through its successful implementation across a number of different technology platforms. We further validate the approach through replication of a published clinical study examining racial disparities in oxygen therapy, confirming its feasibility for reproducing complex analytical findings. While the specification has limitations - including single-resource projection constraints and unidirectional transformation - these reflect deliberate design decisions to prioritise simplicity and implementation feasibility over maximum expressivity.

## Results

### Specification

In designing the view definition specification^19^, the key goal was to provide a standardised way to extract data from FHIR resources into tabular structures while preserving FHIR semantics and conventions. By keeping the specification focused and implementation-friendly, view runners can be easily developed for different data processing platforms, allowing the same view definitions to be executed across diverse toolchains. This approach eliminates the need for analysts to write custom FHIR transformation code for each use case - they can instead rely on reusable view definitions and platform-specific runners to integrate FHIR data into their existing workflows.

We deliberately focused on addressing only the FHIR-specific aspects of data projection. General data manipulation capabilities that are widely available within the tools that users already have were specifically excluded from the scope, including:

1. Joining of views
2. Sorting of rows
3. Grouping and aggregation of results
4. Limiting the number of results

**Fig. 1:**
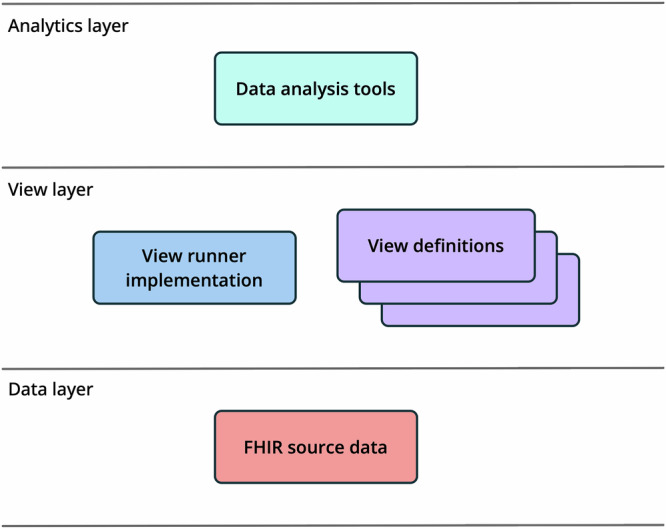
Architectural layers. Overview of the conceptual architecture for SQL on FHIR. The data layer represents FHIR data sources, including FHIR servers, NDJSON files, and FHIR data held within databases. The view layer uses view definitions to define table-like views of the data, and uses view runners to execute these definitions. The analytics layer consumes the tabular data produced by the views as part of analysis tasks. This diagram is reproduced with permission from the SQL on FHIR Working Group.

The view definition specification was designed to play an enabling role within a larger conceptual architecture that consists of three layers: a data layer, a view layer, and an analytics layer (see Figure 1). The data layer consists of some representation of FHIR data. The view layer consists of view definitions, and view runner implementations that can execute these definitions over the FHIR data. The analytics layer consists of tools and platforms that can consume the output of the view runners and perform analytics on the data.

The data layer can accommodate various FHIR representations, including FHIR servers implementing the REST or Bulk APIs, FHIR NDJSON (Newline-Delimited JSON) files in object storage systems, or FHIR data mapped to relational database schemas. The flexibility in how FHIR data can be stored and queried is handled by the view runner implementations, which encapsulate the specific assumptions and requirements of each representation.

View definitions are designed to be agnostic to these implementation details, allowing them to be portable across different view runners without modification. View definitions have the potential to be reusable across use cases that share common data requirements, and a suitable view runner can be selected to execute them based on the specific requirements of the analytic platform. View definitions that cover the data requirements of common use cases could be published as part of FHIR implementation guides to make it easier for users to consume FHIR data.

The name ‘SQL on FHIR’ emphasises the use of SQL as a common analytical approach. There are many different dialects of SQL with their own implementation-specific features, and the view definition addresses this by delivering FHIR data as a simple tabular output that can be queried using a very small subset of the SQL language. This makes it easier to write SQL queries that are portable across different SQL implementations.

Transpilation of view definitions to SQL or other query languages is attractive from a performance perspective, as it allows for the resulting query to be combined with other queries within the analytic layer and optimised by the query engine as a whole.

While SQL is a well-supported approach for querying views within the analytic layer, the view definition does not assume its use. The view definition can act as a bridge between the data layer and anything that can consume tabular data, including data science languages like R and Python, spreadsheet tools such as Microsoft Excel, business intelligence tools and training tasks for machine learning models.

A view is defined using:

1. At least one ‘select’ directive, which describes a tabular projection of a FHIR resource
2. Any number of ‘where’ directives, which specify a set of inclusion criteria

Each select directive produces its own tabular result. Each of the ‘select’ results is combined into a single result using a cross-join (Cartesian product).

Each ‘select’ directive is defined using the following sub-directives:

1. ‘column’
2. ‘forEach’
3. ‘forEachOrNull’
4. ‘unionAll’
5. ‘select’ (nested)

The following sections contain examples that are based upon executing view definitions over some example FHIR R4 resources. These resources are represented by JSON files that have been attached to this document as Supplementary materials.

Each column within a view is defined using a ‘column’ directive, which consists of a name and a FHIRPath expression. FHIRPath^20^ is a language that provides a simple way to select values and predicates from FHIR resources. The evaluation of a FHIRPath expression against a FHIR resource returns a collection of values that can be used to populate a column within the view.

For example, Listing 1 shows a view definition selects the patient identifier and birth date from a set of Patient resources:

#### Listing 1

‘column’ example 

This would produce Table 1 with two columns - an ‘id’ column containing the resource identifier, and a ‘birth_date’ column containing the patient’s date of birth, for example:

**Table 1:** ‘column’ result

id	birth_date
patient-1	1980-01-01
patient-2	1992-07-15
patient-3	1965-12-31

A FHIRPath expression that is querying a FHIR resource can return multiple values when traversing repeating elements. When defining a column, you can assert that the expression returns at least one value by setting the ‘collection’ element to ‘true’. This will result in an array rather than a scalar value within the column.

If the ‘collection’ element is set to ‘false’ (or omitted), then the expression must only ever return a single value. If the expression returns more than one value, the view runner will report an error.

When FHIRPath expressions are expected to return multiple values, the ‘forEach’ directive can be used to generate a row for each value returned.

An expression is passed to the ‘forEach’ directive that describes the collection that should be unnested. A set of ‘column’ directives within the containing ‘select’ is used to describe the columns that will be generated for each value, relative to the expression that is being unnested.

If the ‘forEach’ expression evaluates to an empty collection (i.e., no rows will be generated) the parent resource will be omitted from the result entirely. This is similar to the behaviour of an ‘INNER JOIN’ in SQL.

For example, the view definition in Listing 2 will generate a row for each Patient address:

#### Listing 2

‘forEach’ example 

If a Patient has three different addresses recorded, this view will generate three rows - one for each address (see Table 2). A Patient with no addresses recorded would be omitted from the results entirely.

**Table 2:** ‘forEach’ result

id	address_line	address_city	address_state	address_postal_code
patient-1	1 Main St	Springfield	IL	62701
patient-1	42 Second Ave	Chicago	IL	60601
patient-1	789 Third St	Peoria	IL	61602
patient-3	42 Second Ave	Chicago	IL	60601

The ‘forEachOrNull’ directive is a variation on ‘forEach’ that will always generate at least one row per resource. The row will be populated with a null value if the result of evaluating the expression was an empty collection. This is similar to the behaviour of an ‘OUTER JOIN’ in SQL.

For example, we modify the previous view to use ‘forEachOrNull’ instead of ‘forEach’ in Listing 3:

#### Listing 3

‘forEachOrNull’ example 

A Patient with no addresses would now appear in the results with null values for the address fields (see Table 3).

**Table 3:** ‘forEachOrNull’ result

id	address_line	address_city	address_state	address_postal_code
patient-1	1 Main St	Springfield	IL	62701
patient-1	42 Second Ave	Chicago	IL	60601
patient-1	789 Third St	Peoria	IL	61602
patient-2	null	null	null	null
patient-3	42 Second Ave	Chicago	IL	60601

The ‘forEach` and ‘forEachOrNull` directives provide a way of handling deeply nested data within FHIR resources. For example, a clinical observation might contain multiple component observations, each with multiple coding systems and reference ranges. The ability to nest ‘select` statements with multiple ‘forEach` or ‘forEachOrNull` directives allows precise control over how these structures are flattened into tabular form, while also preserving important correlations between the data elements.

The ‘unionAll’ directive is used to combine the results of two or more selections into a single set of columns. Each selection within a ‘unionAll` must have the same column names. This directive can be useful for containing a unified view of information that is collected from multiple different areas of a FHIR resource.

For example, the view definition in Listing 4 combines the patient’s identifier and name with their address information:

#### Listing 4

‘unionAll’ example 

This view will produce a table with the patient’s identifier, name, family name, and contact phone information (see Table 4).

**Table 4:** ‘unionAll’ result

id	name	family	contact_phone
patient-1	Aisha	Khan	555551234
patient-1	Aisha	Khan	555559876
patient-2	Juan	Rodriguez	555542233
patient-3	Wei	Zhang	555554321
patient-3	Wei	Zhang	555555678

The ‘where’ directive is used to filter the set of resources that are included in the view. A resource will only be included in the view if all the expressions in the ‘where’ directive evaluate to true.

For example, the view definition in Listing 5 will include only Patients who have an active status.

#### Listing 5

‘where’ example 

This view will produce a table with only the active patients (see Table 5).

**Table 5:** ‘where’ result

id	given_name	family_name
patient-1	Aisha	Khan
patient-3	Wei	Zhang

A view definition operates on a single FHIR resource type only. Rather than attempt to define join semantics within the view definition itself, the specification defines two new FHIRPath functions that serve as an abstraction for the generation of join keys.

The ‘getResourceKey’ function is used to generate a key that uniquely identifies a resource within a FHIR data set. The ‘getReferenceKey’ function is used to generate a foreign key that can be used to join a resource to another resource that it refers to.

Any two views that refer to each other using resource references can be combined using an equijoin on the keys generated by these functions.

To demonstrate how to join a Patient view with an Encounter view using the ‘getResourceKey’ and ‘getReferenceKey’ functions, we will define two view definitions (Listings 6 and 7) and show the results of executing them individually (Tables 6 and 7). Then, we will provide a simple SQL example (Listing 8) to create a single joined view (Table 8).

**Table 6:** Join example — Patient view result

patient_id	name	family
patient-1	Aisha	Khan
patient-2	Juan	Rodriguez
patient-3	Wei	Zhang

**Table 7:** Join example — Encounter view result

encounter_id	patient_id	encounter_date
encounter-1	patient-1	2023-01-01
encounter-2	patient-2	2023-02-15
encounter-3	patient-1	2023-03-10

**Table 8:** Join example — Final joined result

patient_id	name	family	encounter_id	encounter_date
patient-1	Aisha	Khan	encounter-1	2023-01-01
patient-1	Aisha	Khan	encounter-3	2023-03-10
patient-2	Juan	Rodriguez	encounter-2	2023-02-15

#### Listing 6

**Join example**- Patient view definition 

#### Listing 7

**Join example**- Encounter view definition 

#### Listing 8

**Join example** — SQL to create a joined view

SELECT

 p.patient_id,

 p.name,

 p.family,

 e.encounter_id,

e.encounter_date

FROM

PatientView p

JOIN

EncounterView e

ON

p.patient_id = e.patient_id;

The implementation details of the ‘getResourceKey’ and ‘getReferenceKey’ functions are purposefully left unspecified. This allows view runner implementations to provide custom methods for generating these keys that are appropriate for the execution context within which they are intended to operate.

For example, a view runner might support absolute URL references and the fetching of referenced resources from a FHIR server within a trusted source environment. Another view runner might support the processing of resources that contain other resources that do not have their own independent identity.

These functions, in conjunction with the other directives in the view definition, complete the set of tools required to work with complex clinical data that involves relationships between multiple resource types. Consider a clinical scenario requiring analysis of patient encounters, their associated procedures, and medication administrations. While deeply nested structures within a the resources are handled by ‘forEach’ directives, relationships across resources (such as medications administered during a procedure performed in an encounter) are facilitated by these join functions.

**Fig. 2:**
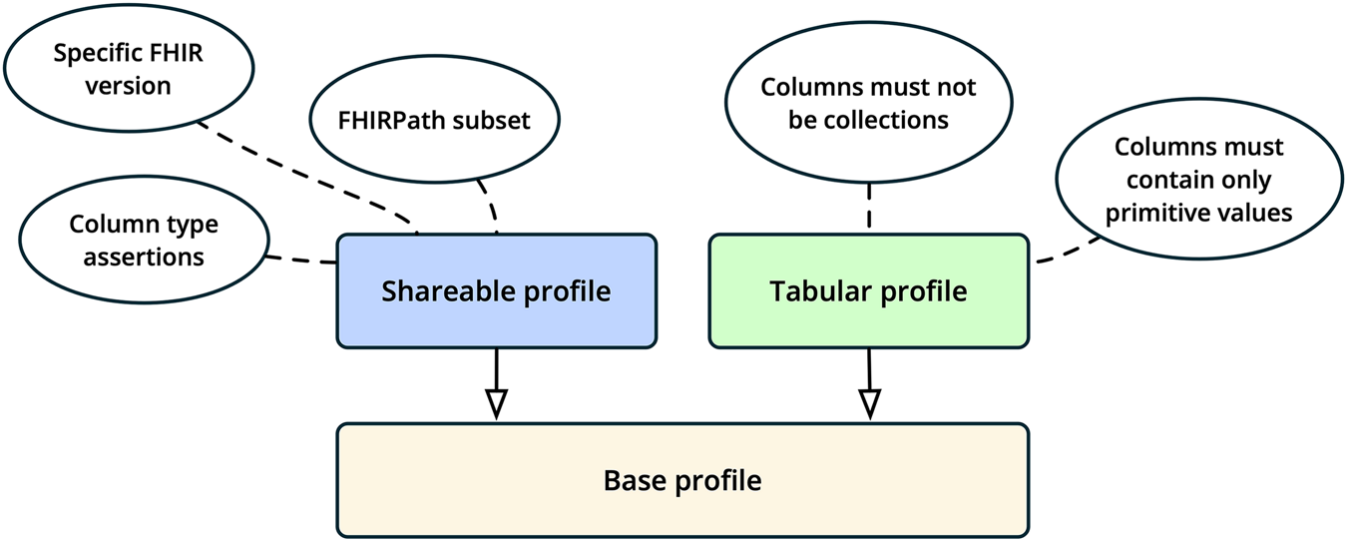
View definition profiles. Profiles defined as part of the SQL on FHIR view definition specification. The base profile has minimal requirements for quick, on-demand use. The shareable profile adds rules for identifying resources, versioning, and limits FHIRPath usage to work better across different systems. The tabular profile further restricts column data types to simple values only, making the data compatible with formats like CSV and systems that cannot handle complex data.

A profile architecture has been created to allow for constraints to be progressively applied to view definitions based upon their intended use (see Fig. 2).

The ‘base’ profile is automatically applied to all view definitions and has almost no constraints, which is appropriate for a view that is authored and executed on-demand. Views that conform to the base profile are free to define columns that contain complex data types such as arrays and objects, which may be desirable when the consuming query environment supports subsequent querying of these types.

The ‘shareable’ profile^21^ imposes some requirements that relate to the identification and versioning of a view definition that is intended to be shared and have a publication lifecycle. It also requires declaration of the FHIR version that the view is intended to be used with, and the FHIR type of each column. This is to ensure that the view can be executed consistently across different implementations.

The ‘shareable’ profile also defines a FHIRPath subset that is used to constrain the level of FHIRPath functionality that is required to be supported by the view runner. This lowers implementation complexity and improves the interoperability of views that declare this profile.

The ‘tabular’ profile^22^ adds the requirement that all columns must contain primitive data types only. This is to ensure that the result of the view can be represented as a simple table, which is necessary for many tools and formats such as CSV.

A view can assert the set of profiles that it conforms to using the ‘meta.profile’ element, e.g., both ‘shareable’ and ‘tabular’.

### Implementations

A number of different implementations of the view definition specification were developed alongside the authoring of the specification. These implementations were developed independently and were used to validate the correctness and feasibility of the specification. The implementations were each tested against a common set of test cases that have been published as part of the specification.

The reference implementation of a view runner was developed in JavaScript and is freely available as part of the SQL on FHIR specification. This implementation is designed to be simple and easy to understand and is intended to be used as a reference for other implementations.

Pathling^17^ is an open-source project that developed a view runner that is capable of converting a view into an Apache Spark^23^ SQL query. This implementation is designed to be used in a production environment and can execute views over large datasets of Newline-Delimited JSON (NDJSON), FHIR Bundles and streaming sources.

Aidbox^24^ is a commercial FHIR server that now has a view runner that is capable of executing views over a FHIR dataset stored in a PostgreSQL^25^ database. These views can be created and optionally materialised within the database through the FHIR API.

Google’s Open Health Stack initiative implemented a view runner as part of their FHIR Data Pipes project^26^. This implementation has an emphasis on its ability to be deployed over a wide variety of data warehouse technologies.

Dan Gottlieb created a view runner based on DuckDB called FlatQuack^27^. It can execute views over FHIR data from a variety of sources and transform it efficiently using an in-process SQL engine.

Medplum^28^ is an open-source FHIR application platform that has implemented a view runner in TypeScript, which is available through their developer API.

A view runner has been implemented within the official HL7 implementation guide publisher^29^. It is capable of validating view definitions and using them to build tables within an internal database based on FHIR resources contained within the implementation guide. Authors can write SQL queries within their page templates that query this database and render the results as tables within pages in the published content.

### Study replication

To demonstrate the feasibility of the view definition specification, we replicated a study by Gottlieb, et al.^30^ using views over a FHIR rendition of the MIMIC-IV dataset^31,32^. The study examines differences in supplemental oxygen administration between patients of different races and ethnicities.

This exercise was conducted with two main goals:

1. To demonstrate that it is feasible to use a combination of views and SQL to replicate the results of the original study.
2. To demonstrate that the views and SQL queries can be run across multiple diverse view runners and database implementations and produce the same results.

The process of executing the views and the associated SQL queries was repeated for two different combinations of view runner and SQL implementation:

1. Pathling and Apache Spark SQL.
2. Aidbox and PostgreSQL.

**Fig. 3:**
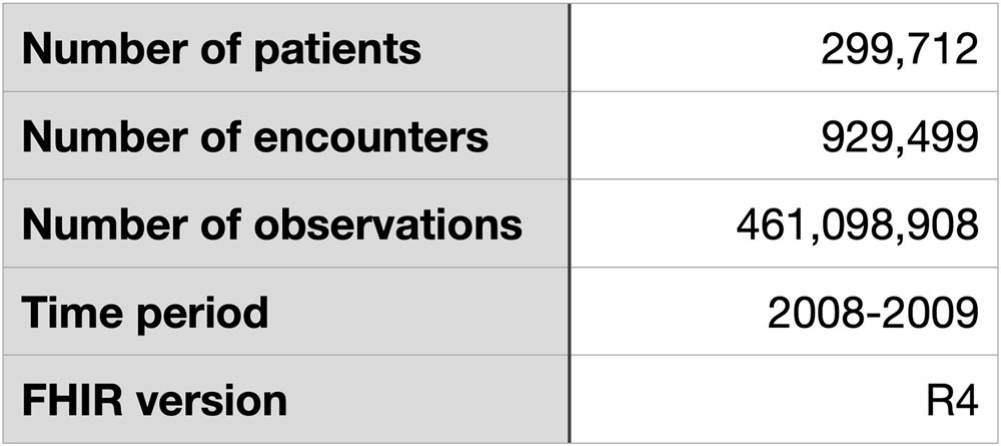
Snapshot of MIMIC-IV on FHIR dataset used in evaluation. Size and composition of the dataset used for testing. The dataset contains 299,712 patient records, 929,499 clinical encounters, and 461,098,908 observations from 2008 to 2019, all following the FHIR R4 standard. This provides sufficient data to test how well the SQL on FHIR approach works for preparing large clinical datasets for analysis.

See Fig. 3 for a snapshot of the MIMIC-IV on FHIR dataset used in the evaluation.

The analysis showed that the same results could be obtained from both implementations using the same view definitions and SQL queries. This demonstrates that the data requirements for a real-world use case can be represented in a standard way that can be exchanged and executed across different implementations.

The results also confirmed the key finding from the original study, that supplemental oxygen administration is significantly lower for patients of Asian, Black and Hispanic ethnicities as compared to White patients. Specifically, our analysis found that Asian patients were 22% less likely, Black patients were 19% less likely, and Hispanic patients were 15% less likely to receive supplemental oxygen compared to White patients with similar clinical presentations (*p* < 0.001 for all comparisons).

### Review and comparison with other approaches

We reviewed a number of other approaches to determine their suitability for application to the stated problem, and to evaluate how they compare with the view definition specification. This includes some that have been conceived to solve the same problem and others that are much more general in nature or inhabit a related but overlapping problem space.

The initial version of the SQL on FHIR^15^ specification took a different approach to the problem of transforming FHIR for analytic tasks. The original specification described a method of querying FHIR data that involved using ANSI SQL^33^ over a logical representation of the FHIR data model. The FHIR data model includes nested structures and repeating fields, and this approach required the use of SQL language features such as UNNEST and the ‘dot operator’ to access these fields.

This approach works well for analytic workloads when using query engines that both support this syntax and can execute it efficiently. However, there are two problems with it:

1. The syntax defined for accessing arrays and nested structures in SQL has not achieved a good level of standardisation within implementations, making it difficult to write SQL queries that are portable.
2. SQL users are generally less familiar with the syntax used to traverse and unnest complex nested data structures.

The view definition specification addresses these challenges by making less assumptions about the underlying data representation. This makes it simpler to implement over a wide range of data sources.

Pathling^17^ implements several view abstractions, and the most analogous to the SQL on FHIR view definition is the ‘Extract’ operation. The ‘Extract’ operation also takes a FHIR resource type and a set of FHIRPath expressions as input and produces a tabular result.

One of the differences is that the ‘Extract’ operation automatically unnests repeating fields, rather than requiring the user to describe how they would like the data to be unnested. When an ‘Extract’ operation is carried out on a set of columns that contain common ancestor elements, the nested groupings will be kept together and any invalid combinations eliminated. This works in a similar way to the use of ‘select’ with ‘forEachOrNull’.

The advantage to this approach is that the query is more concise, with the author not being required to explicitly describe the unnesting of repeating fields. The disadvantage is that it lacks the level of fine-grained control that the view definition provides over the unnesting process.

The other difference is that the ‘Extract’ operation supports ‘resolve’ and ‘reverseResolve’ FHIRPath functions for traversing resource references within expressions. This means that the result of an ‘Extract’ operation can include data from multiple resource types. One of the down sides of this is that it makes it difficult to optimise the query, as the possible presence of reference resolution anywhere in the expressions requires careful planning of joins.

CQL^34^ is a domain-specific language and execution model that is used to express clinical quality measures. The CQL language is a superset of FHIRPath and supports additional functions and language constructs. It has a larger scope than the view definition and is designed not only to extract data from FHIR and other data models, but also to represent query logic that can be executed over this data. Although it is possible to use CQL to extract tabular data, it is not the primary purpose for it was designed, and it is generally used to execute pre-defined measures rather than for transforming data for ad-hoc analysis.

CQL provides a comprehensive approach to clinical query authoring and execution that includes the expression of libraries and their dependencies, data dependencies (including terminology dependencies), and the logic required to calculate measures themselves. View definitions provide a solution to a small part of the problem that CQL tackles, but are more generalisable (i.e., they make fewer assumptions about the use case for the data extraction) and are easy to compose with other tools and data sources.

The degree of difficulty for implementing CQL is higher than the view definition, especially if targeting a comprehensive coverage of CQL features. This means that CQL implementations are more costly to build and maintain, and there are only a small number that have achieved good coverage of the specification. CQL authoring also requires a higher level of expertise due to the scope and complexity of the language.

FML^16^ was created within the FHIR specification to solve the problem of mapping between FHIR and other arbitrary data structures. It is a domain-specific language that uses FHIRPath expressions, and it uses the ‘StructureMap’ resource within FHIR as its abstract syntax representation.

As compared to the view definition, FML solves a less constrained problem in that it is capable of mapping FHIR data to any arbitrary data structure. For the case where the user is mapping FHIR to a tabular form, they are required to be more explicit in their description of the mapping than is required for the view definition.

FML has the capability to define embedded concept maps for mapping between concepts in different terminologies. This is a feature that the view definition does not yet have, and this may be desirable for some use cases.

Although FML can be represented in an abstract form within a resource (and therefore in JSON), it is more complicated than the view definition and is not easy to read or easily translated into other forms such as function parameters.

Because FML has a more general applicability than the view definition, it has a higher degree of difficulty of implementation. At the time of writing and to the best knowledge of the authors, there are no FML implementations that directly target the creation of views within databases or other analytic tools.

Recent work by Bossenko et al.^35^ has explored the use of FML for health data transformation in the Estonian National Health Information System, demonstrating its utility for complex transformations while also highlighting the usability challenges.

FQL^18^ was proposed by Firely as a query language designed to project and filter FHIR data into a tabular result, agnostic of the storage implementation. It was an important source of inspiration for the nested selection mechanism (‘select` with ‘forEach’ or ‘forEachOrNull’) in the view definition, through a feature called ‘group unwrapping’.

The goals of FQL and the view definition are quite similar. FQL was initially designed to automate the extraction of data from resources within FHIR implementation guides. This capability has now been added to the official HL7 implementation guide builder using view definitions.

Like the view definition, FQL also has the constraint that a single resource type must be selected and projected. This eliminates the complexity of composing and executing queries that span multiple different resource types. However, FQL does not provide helper functions like ‘getResourceKey’ and ‘getReferenceKey’ to assist with the joining of results.

FQL uses a domain-specific language that incorporates FHIRPath but is structurally similar to SQL. The advantage of this is that it is easy to learn for those who are already familiar with SQL. One downside of this approach is that implementers are required to write a parser for the language, in addition to a FHIRPath parser.

Whistle^36^ is another general-purpose data transformation language that was written by Google for use within the healthcare domain. Whistle provides powerful data path expressions, nested data manipulation capabilities, and various merge modes (merge, replace, append, extend). Its use has been demonstrated by the MENDS-on-FHIR project^37^ as part of the task of transforming an OMOP CDM dataset to FHIR.

Similar to FML, Whistle supports mapping between arbitrary data structures and could satisfy the use case of transforming FHIR into a tabular form. It uses a specialised syntax focused on data paths and transformations, with complexity that scales in proportion to the data being transformed. While Whistle is quite powerful for complex healthcare data transformations, it requires specialised knowledge of its syntax and semantics, making it less accessible to users primarily familiar with SQL or other common query languages.

FhirExtinguisher^38^ provides a simple approach for transforming FHIR data into a tabular form. It is implemented as a server that provides an API for executing these transformations.

Data are retrieved from a configured FHIR server via the FHIR Search API and are transformed into a tabular form using the FHIRPath expressions by the FhirExtinguisher server. The results are returned to the client in CSV format.

Each of the FHIRPath expressions is accompanied by a list processing behaviour directive. The directives ‘join’, ‘explodeWide’, and ‘explodeLong’ are provided to control the way that repeating fields are unnested. This allows for simple string concatenation, unnesting into columns and unnesting into rows, respectively. It does not allow for the same level of control over the unnesting of deeply nested structures as the view definition.

This approach is also limited by its use of the FHIR Search API, which requires transmission of data over network requests and may not be as efficient as directly querying a database containing the FHIR data.

FHIR-PYrate^39^ is a Python package that automates the task of retrieving data from a FHIR server and transforming it into Pandas data frames. It supports FHIRPath as a way of defining data frame columns.

This solution does not provide a way of describing how to flatten or unnest FHIR data, beyond the ability of the user to use the Pandas data frame API to achieve this. Collections returned by FHIRPath expressions are represented as lists within data frame columns, and there is no support for correlating the contents of related lists within different columns.

The performance of FHIR-PYrate is limited by the performance of the requests to the FHIR REST and Search APIs that it uses to retrieve data. This may be a limiting factor when working with large datasets.

The fhircrackr^40^ package is an R-based solution for transforming FHIR resources into tabular data frames suitable for statistical analysis. It addresses the challenge of FHIR’s complex nested structure, which is not directly amenable to statistical processing. The package uses XPath expressions to extract specific elements from FHIR resources and offers multiple strategies for handling repeating elements.

For handling multiple entries in FHIR resources, fhircrackr provides three main approaches: concatenating values with a separator, preserving index information for each entry, or using a ‘melting’ function to spread multi-entry columns across rows. This flexibility gives users control over how to represent complex FHIR data in tabular form, though it requires users to be familiar with XPath expressions and the XML structure of FHIR resources.

Like FHIR-PYrate, fhircrackr retrieves data from FHIR servers via the REST API, which may impact performance when working with large datasets. Its focus on R integration makes it a practical solution for researchers and analysts who work primarily in the R ecosystem, but this specialisation may limit its broader applicability across different environments.

GraphQL^41^ is a query language for APIs, and guidance is provided within the FHIR specification^42^ on how to use GraphQL to query FHIR data. One of the features of the FHIR GraphQL specification is the ‘@flatten’ directive, which can be used to unnest repeating fields within a FHIR resource.

When a repeating element is encountered, the ‘@flatten’ directive will cause the children of that element to become lists within the parent object.

Where the ‘@flatten’ directive differs from the view definition is that its output is a graph (commonly represented as a JSON object), rather than a table. If two repeating elements with a common parent element are flattened into the top-level object, the correlation between the elements within each list is lost. This means that this approach cannot be used to create a tabular result with correlated data from different repeating elements in separate columns.

The SQL/JSON standard^43^ is an extension to the SQL language that provides a set of functions and operators for querying and manipulating JSON data within a relational database. This standard allows for the integration of JSON queries into SQL. Key features of SQL/JSON include the ability to extract values from JSON documents, transform JSON data into relational formats, and perform complex queries using expressions.

The path expression language used within SQL/JSON is based upon JSONPath^44^, which is a language that provides a simple way to select values and predicates from JSON data. It performs a similar role to FHIRPath in terms of extracting data from a JSON document but is more general in its applicability. JSONPath can be used as a way of implementing view definitions within a database that supports it as a way of querying JSON typed columns.

There is also a ‘JSON_TABLE’ function within SQL/JSON that can be used to compose JSONPath expressions into an overall view over a set of JSON data. This functionality is very similar to the view definition and the design is closely aligned.

At the time of writing, the SQL/JSON standard and ‘JSON_ TABLE’ function are supported by PostgreSQL, Oracle^45^ and DB/2^46^. There are some differences in the implementation of the path expression language across these systems.

To enable a more systematic comparison of the various FHIR data transformation approaches, we have developed a scoring framework with four key dimensions, each rated on a 1–5 scale where higher scores represent greater value (see Table 9). For each dimension, we provide scoring criteria to ensure transparency and consistency in our evaluation.

- **Expressivity**: Evaluates the power and flexibility of the transformation language.
  – *1*: Very low expressivity (extremely basic transformations only)
  – *2*: Low expressivity (simple mapping capabilities)
  – *3*: Medium expressivity (can handle common transformation needs)
  – *4*: High expressivity (powerful query capabilities, complex transformations)
  – *5*: Very high expressivity (complete transformation flexibility, including non-tabular target formats)
- **Ease of Implementation**: Assesses how easily the approach can be implemented.
  – *1*: Very difficult (very high level of effort to implement)
  – *2*: Difficult (high level of effort to implement)
  – *3*: Moderate (requires moderate implementation effort)
  – *4*: Easy (straightforward implementation)
  – *5*: Very easy (trivial to implement)
- **Portability**: Measures how easily the approach can be used across different systems.
  – *1*: Very low portability (tightly coupled to one specific implementation)
  – *2*: Low portability (coupled to a small number of technologies that share similar characteristics)
  – *3*: Medium portability (could be implemented across broad range of technologies, but only with limitations or a high degree of effort)
  – *4*: High portability (could be implemented across a broad range of diverse technologies with a moderate degree of effort)
  – *5*: Very high portability (demonstrated to be effective and practical across a broad range of diverse technologies)
- **Ease of Use**: Evaluates how approachable the approach is for users.
  – *1*: Very difficult to use (requires deep expertise)
  – *2*: Difficult to use (significant learning curve)
  – *3*: Moderate ease of use (requires a higher level of effort or relies upon a significant level of specialised knowledge)
  – *4*: Easy to use (approachable for most technical users)
  – *5*: Very easy to use (minimal learning required)

**Table 9:** Comparison of FHIR data transformation approaches using a 1–5 scale

Approach	Expressivity	Ease of Implementation	Portability	Ease of Use	Total
SQL on FHIR v2	3	4	5	4	16
SQL on FHIR v1	4	4	3	3	14
FQL	3	3	4	4	14
SQL/JSON	4	3	4	3	14
Pathling Extract	3	3	3	4	13
FHIR/GraphQL	2	4	4	3	13
Whistle	5	2	3	2	12
FML	5	2	3	2	12
FHIR-PYrate	2	4	2	4	12
FhirExtinguisher	1	4	4	3	12
fhircrackr	3	3	2	3	11
CQL	5	1	3	2	11

Note that this evaluation is done in the context of determining an approach that can be used as a standard, implementation-agnostic method for transforming FHIR data into tabular form. The evaluation is not intended to be interpreted as a reflection on the value that these approaches provide as standalone solutions or the value that has been contributed through their development.

## Discussion

The design of the view definition specification incorporates several assumptions:

- Existing query engines and analytics platforms provide sufficient capabilities for data manipulation once FHIR data is transformed into tabular form. This justifies the exclusion of features like sorting, grouping, and join operations from the specification.
- Users primarily require read-only access to FHIR data for analytics, making the unidirectional nature of the transformation acceptable.
- The benefits of simplified implementation and greater portability outweigh the limitations imposed by the single-resource constraint.
- FHIRPath expressions, even when limited to a subset of the language, provide sufficient power to address common data extraction needs.
- Users are willing to invest time in learning the view definition structure and FHIRPath syntax, or that appropriate tooling can be developed to mitigate this burden.

By limiting the scope of functionality to the minimum necessary for the projection of FHIR data, users are free to use the workflows that they are already familiar with to perform the actual analysis. This separation of FHIR-specific transformation from general data manipulation operations allows integration with existing analytic infrastructure and leverages capabilities present in SQL and other data processing systems. This separation was demonstrated in the study replication, where view definitions combined with standard SQL successfully reproduced the findings of the original clinical study and were able to be executed consistently by two different implementation stacks.

The utilisation of FHIRPath as an existing standard eliminates the need for a new query language, which allows implementations to leverage existing parsing and evaluation engines. FHIRPath is already to known to many people who use FHIR for interoperability applications, and its complexity for newcomers is mitigated by the use of a small subset of the language within the view definition specification. However, the requirement for users to be familiar with both FHIRPath and the view definition structure may still present a barrier to entry. Potential mitigations include the development of higher-level tooling and the reuse of published view definitions.

The single-resource constraint, though limiting expressivity, simplifies implementation and improves portability. By limiting each view to a single resource, we remove assumptions about how views will be combined by downstream processing, which could include dynamic methods for joining tables in addition to more traditional SQL joins. The ‘getResourceKey’ and ‘getReferenceKey’ functions provide a powerful abstraction for view runners to implement custom join logic, enabling use case-specific optimisations.

The profile architecture provides graduated constraints based on intended use, maintaining flexibility for ad-hoc applications while enabling standardisation for shared views. By organising the constraints within the specification into optional layers, we can maximise the breadth of possible applications while still enabling distribution and interchange.

One of the challenges identified during the evaluation was inconsistent column type handling across implementations, requiring an additional type-casting step when executing SQL queries. This indicates a need for standardised type hints, and this could be addressed through the use of the ‘tag` element in conjunction with ANSI SQL type identifiers. This is currently a suggestion within the specification, but it may be beneficial to make this a requirement in future versions.

The systematic comparison of the approaches in Table 9 suggests an inverse relationship between expressivity and implementation complexity. Approaches with high expressivity, such as CQL, FML, and Whistle, provide extensive transformation capabilities but require significant implementation effort and specialised knowledge to use them effectively. In contrast, the SQL on FHIR v2 specification, while more constrained in expressivity, demonstrates higher portability and easier implementation. This seems to validate the design philosophy of applying deliberate constraints to a well-defined problem space rather than addressing all possible data transformation scenarios. The existence (at the time of writing) of eight independent implementations using a diverse set of technologies provides further support for this.

The development and evaluation process yielded several observations that may inform similar standardisation efforts:

1. It is important to consider how a specification will interact with the existing tools and workflows that are used by its target user group.
2. Utilisation of existing standards may help to ease adoption by leveraging existing knowledge and tools.
3. An open, collaborative development process can foster a high level of community engagement.
4. Reference implementations and test suites ensure consistent interpretation and aid development.

The view definition work intersects with a number of other research streams in health data analytics and interoperability. Cumulus^47^ focuses on federated population-level sharing through FHIR APIs and could potentially utilise view definitions for generating standard tabular representations. The quEHRy system^48^ demonstrates natural language interfaces for FHIR data, which might benefit from operating over standardised tabular views rather than complex FHIR structures. EHRMamba^49^ transforms FHIR data into specialised representations for machine learning, an area where standardised view definitions could provide consistent features. European initiatives such as SmartCHANGE^50^, beHEALTHIER^51^, and CrowdHEALTH^52^ have developed domain-specific analytical frameworks that could potentially utilise the view definition approach as a standardised data preparation method.

The emergence of LLM-based approaches to FHIR analytics, as demonstrated by ALMutairi et al.’s FHIRViz platform^53^, represents a complementary direction to the tabular transformation methods we’ve examined. FHIRViz leverages large language models and knowledge graphs to enable natural language querying of FHIR data with direct visualisation output. While our view definition specification focuses on providing standardised, implementation-agnostic methods for creating tabular structures, the LLM approach prioritises ease of use through natural language interaction. These approaches need not be mutually exclusive; future work could explore combining the precision and portability of standardised view definitions with the intuitive interface of LLM-based systems. For instance, LLMs could potentially be used to generate view definitions based on natural language requests, or to provide more accessible query interfaces to tabular data produced through our approach.

Beyond academic research, the view definition specification has potential applications across multiple healthcare domains. In clinical research, standardised views could facilitate consistent data extraction for multi-centre trials. Within public health, views could help to standardise surveillance reporting across jurisdictions. Healthcare administrators could apply the approach to quality measure calculations and resource utilisation analysis. For pharmaceutical research, the methodology could provide a mechanism for consistent real-world evidence generation across disparate data sources.

There are a number of applications envisioned for the view definition specification that are the subject of current and future work. One of these applications is the use of view definitions to augment the FHIR Bulk Data Access API^54^ to enable the exchange of customised data extracts between systems.

One approach is to define a new operation that allows for the requesting of a bulk data extract using a view definition. This would rely upon the server to have the capability to execute the view definition and return the result in a tabular form.

Another approach is to transpile the view definition into a set of FHIR Search API criteria and required elements that can be passed to the existing Bulk Data Access API. This would not require any changes to existing bulk server implementations and could serve to make bulk extracts more efficient to generate by constraining their content. The consumer of the extract could then implement a view runner to process the result into the desired tabular form.

The working group has also commenced work on the creation of a specification for exchanging FHIR data in Parquet format^55,56^. This would provide an additional option for representing bulk FHIR data that would be more efficient to transmit and transform than NDJSON.

In conjunction with view definitions and the proposals relating to bulk data access, this could enable a wide range of existing analytic tools to more easily consume FHIR bulk data and transform it for onward processing.

The application of view definitions to the task of mapping FHIR data to the OMOP CDM^1^ is also an area of interest to the working group. This has the potential for a FHIR to OMOP mapping to be defined in an implementation-independent way, as a combination of view definitions and SQL queries.

The possible application of view definitions to other standards such as HL7 v3 is also another area that could be explored. The working group is open to collaboration with other communities that are interested in the use of view definitions to support their own interoperability and analytic needs.

## Methods

### Working group process

The ‘SQL on FHIR Working Group’ is a group of individuals that have an interest in solutions relating to FHIR analytics. The group has an open process that allows for anyone to contribute and provide feedback into specification development. There is an open invitation for participation and recordings of all meetings are made available to the public. All specifications and software produced by the group are released into the public domain via the Creative Commons CC0 and MIT licences.

The group met weekly over the course of 18 months to develop the view definition specification. Attendees of the meetings brought diverse experience with FHIR analytics from clinical, research, and commercial aspects, enabling the collection of real-world use cases across multiple domains. Common patterns and requirements emerged through sharing and discussion, and we were subsequently able to identify the most promising candidate approach and agree upon a method for validating it.

As part of the process, the group also presented their work at HL7 FHIR Connectathon events and at the FHIR DevDays conference to promote feedback and discussion within the broader FHIR community.

### Testing and implementation

The process for developing the specification was driven by multiple different independent implementations of the proposal. A reference implementation was also collaboratively developed by the working group to accompany the specification and help communicate its semantics. This process helped to ensure that the resulting specification was implementable and ensure that a diverse set of use cases was considered.

A test suite was developed to verify the behaviour of the reference implementation and other implementations. At the time of publication, eight different independent implementations were publishing the results of the execution of the test suite. The practice of maintaining a test suite was found to be valuable in ensuring consistency in the interpretation of the specification across different implementations.

### Study replication methodology

The original study^30^ was based on the original MIMIC-IV dataset. The MIMIC-IV on FHIR dataset is derived from MIMIC-IV and enables the same analysis to be demonstrated in a scenario where FHIR source data is available. We received approval from the CSIRO Health and Medical Human Research Ethics Committee to use the MIMIC-IV on FHIR dataset for this purpose (2024_054_LR).

**Fig. 4:**
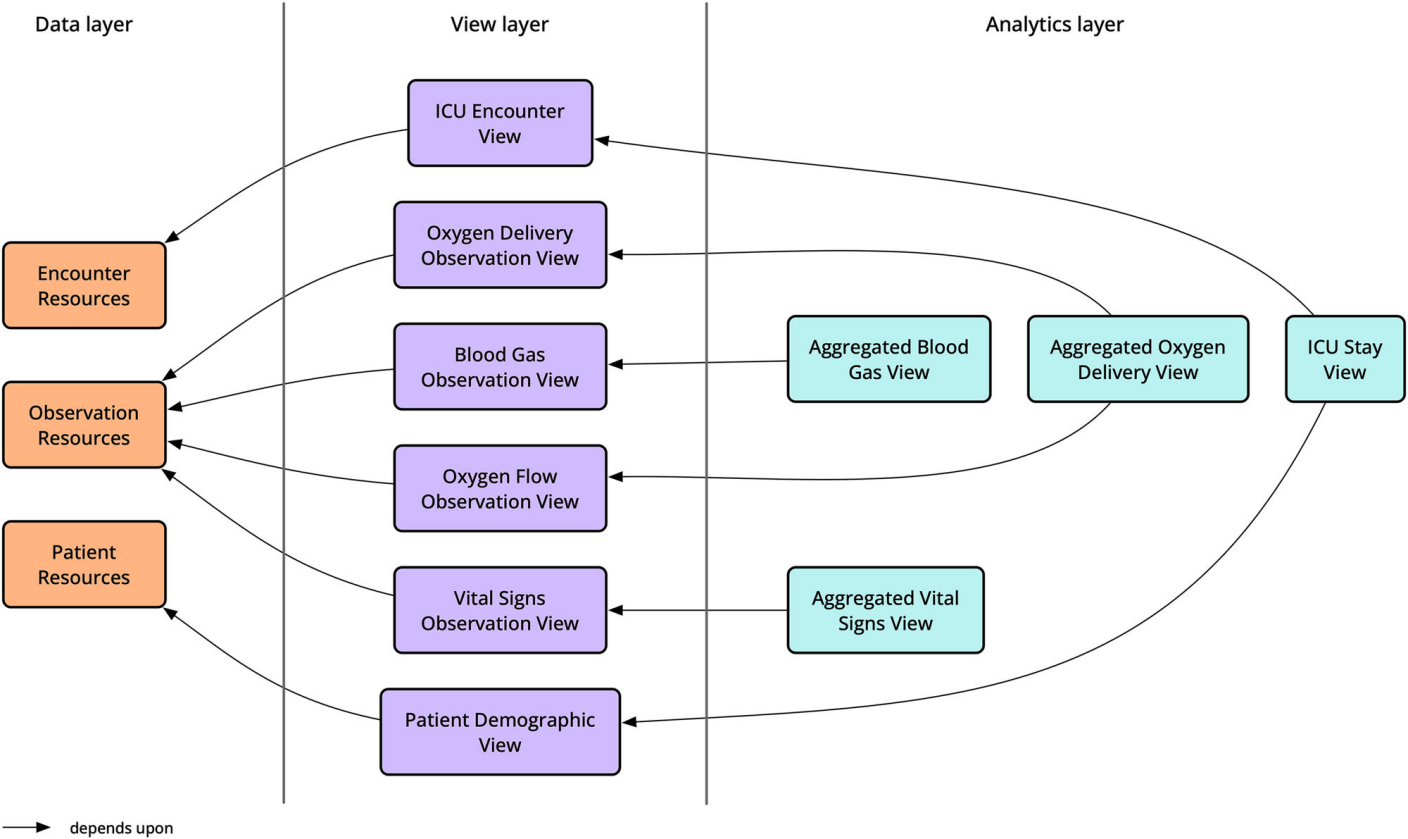
View definitions and compositional queries used in evaluation. The raw FHIR resources were transformed into a set of intermediate views, based on demographics, encounter details and different types of observations. These views were then combined using SQL queries to produce the final set of input variables required for the analysis.

View definitions were used to create an initial level of resource-specific views. SQL queries were then used to combine and transform these views into the final set of input variables that were required to replicate the findings within the original study (see Fig. 4).

**Fig. 5:**
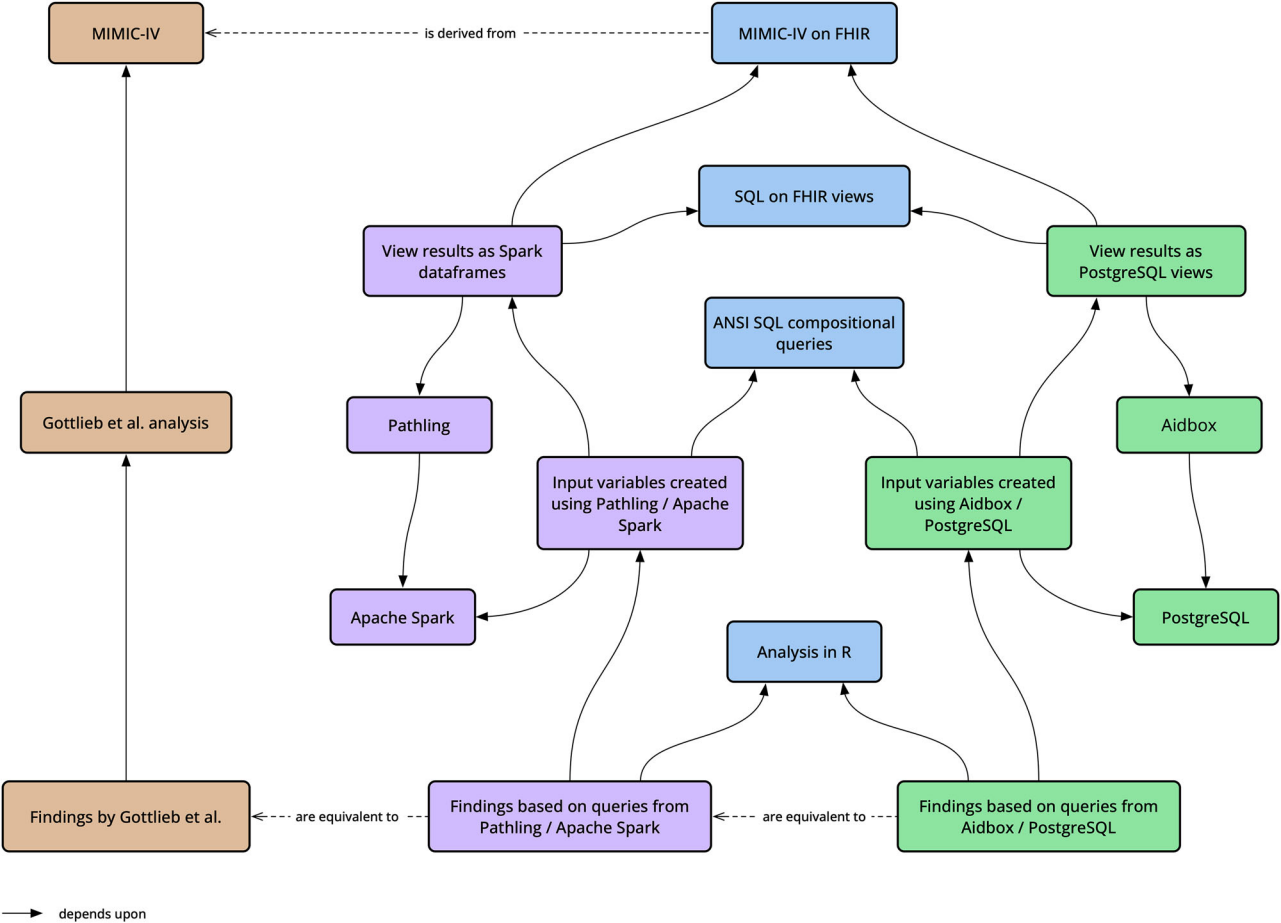
Evaluation of SQL on FHIR views. Approach used to evaluate the use of SQL on FHIR views for replicating the findings of the original study. The view definitions were executed using two different view runners (Pathling and Aidbox), producing two sets of intermediate views. Then the same SQL queries were executed against both sets of views, producing two sets of input variables. Finally, the same analysis pipeline was executed against both sets of input variables, producing two sets of results. The results were then compared to each other and to the original results.

A process was developed to use two different view runners and SQL implementations (Pathling and Aidbox) to separately execute both the view definitions and the SQL queries (see Fig. 5). The results were then utilised within a common analysis pipeline written in R. The analysis pipeline was designed to be agnostic of the underlying data platform and was able to be executed unaltered against the results of both implementations. This approach allowed us to verify that consistent results were produced using both sets of processed data.

During our evaluation, we encountered challenges with data quality and consistency in the MIMIC-IV on FHIR dataset. While we did not need to perform extensive data cleaning for our specific use case, real-world implementations would benefit from approaches like those described by Mavrogiorgos et al.^57^ for healthcare data cleaning. Additionally, considerations around bias in healthcare data, as discussed by Khasawneh and Khasawneh^58^, are important when using view definitions for research purposes.

See the Code availability section for details on how to access the code used in this evaluation.

## Supplementary information

Supplementary information

## Data Availability

The data that was used to produce the evaluation results in this paper is available at https://physionet.org/content/mimic-iv-fhir/2.1/.

## Code availability

The source code that was used to produce the evaluation results in this paper is available at https://data.csiro.au/collection/csiro:63878. This repository contains the view definitions, SQL queries, and analysis scripts used to replicate the study on oxygen administration disparities. The code includes detailed documentation to enable other researchers to reproduce our findings or adapt the approach for their own studies.
