# Supplementary material for: SQL on FHIR - Tabular views of FHIR data using FHIRPath
Source: NPJ Digit Med. 2025 Jun 9;8:342. doi: 10.1038/s41746-025-01708-w (PMC12149319; doi:10.1038/s41746-025-01708-w)
Supplement: Supplementary file 1 — Supplementary information [file 41746_2025_1708_MOESM1_ESM.pdf]

SQL on FHIR — Tabular views of FHIR data using FHIRPath

Supplementary information

Example FHIR resources

encounter-1.Encounter.json

```json
{
  "resourceType": "Encounter",
  "id": "encounter-1",
  "subject": {
    "reference": "Patient/patient-1"
  },
  "period": {
    "start": "2023-01-01"
  }
}
```

encounter-2.Encounter.json

```json
{
  "resourceType": "Encounter",
  "id": "encounter-2",
  "subject": {
    "reference": "Patient/patient-2"
  },
  "period": {
    "start": "2023-02-15"
  }
}
```

encounter-3.Encounter.json

```json
{
  "resourceType": "Encounter",
  "id": "encounter-3",
  "subject": {
    "reference": "Patient/patient-1"
  },
  "period": {
    "start": "2023-03-10"
  }
}
```

patient-1.Patient.json

```
{
  "resourceType": "Patient",
  "id": "patient-1",
  "active": true,
  "name": [
    {
      "family": "Khan",
      "given": [
        "Aisha"
      ]
    }
  ],
  "birthDate": "1980-01-01",
  "address": [
    {
      "line": [
        "1 Main St"
      ],
      "city": "Springfield",
      "state": "IL",
      "postalCode": "62701"
    },
    {
      "line": [
        "42 Second Ave"
      ],
      "city": "Chicago",
      "state": "IL",
      "postalCode": "60601"
    },
    {
      "line": [
        "789 Third St"
      ],
      "city": "Peoria",
      "state": "IL",
      "postalCode": "61602"
    }
  ],
  "telecom": [
    {
      "system": "phone",
      "value": "555551234"
    }
  ],
  "contact": [
    {
      "relationship": [
        {
          "coding": [
            {
```

```
              "system": "http://terminology.hl7.org/CodeSystem/v2-0131",
              "code": "N"
            }
          ]
        }
      ],
      "telecom": [
        {
          "system": "phone",
          "value": "555559876"
        }
      ]
    }
  ]
}
```


patient-2.Patient.json


```
{
  "resourceType": "Patient",
  "id": "patient-2",
  "active": false,
  "name": [
    {
      "family": "Rodriguez",
      "given": [
        "Juan"
      ]
    }
  ],
  "birthDate": "1992-07-15",
  "telecom": [
    {
      "system": "phone",
      "value": "555542233"
    }
  ]
}
```


patient-3.Patient.json


```
{
  "resourceType": "Patient",
  "id": "patient-3",
  "active": true,
  "name": [
    {
      "family": "Zhang",
      "given": [
        "Wei"
      ]
```

```
    }
  ],
  "birthDate": "1965-12-31",
  "address": [
    {
      "line": [
        "42 Second Ave"
      ],
      "city": "Chicago",
      "state": "IL",
      "postalCode": "60601"
    }
  ],
  "telecom": [
    {
      "system": "phone",
      "value": "555554321"
    },
    {
      "system": "phone",
      "value": "555555678"
    }
  ]
}
```
